# Association Between HDL2-C and HDL3-C with Cardiovascular Disease: A Nested Case-Control Study in an Iranian Population

**DOI:** 10.5812/ijem-141550

**Published:** 2023-12-30

**Authors:** Abdolreza Chary, Maryam Tohidi, Mitra Hasheminia, Melika Golmohammadi, Reza Haji Hosseini, Mehdi Hedayati, Fereidoun Azizi, Farzad Hadaegh

**Affiliations:** 1 Department of Biology, Payame Noor University, Tehran, Iran; 2 Prevention of Metabolic Disorders Research Center, Research Institute for Endocrine Sciences, Shahid Beheshti University of Medical Sciences, Tehran, Iran; 3 Cellular and Molecular Endocrine Research Center, Research Institute for Endocrine Sciences, Shahid Beheshti University of Medical Sciences, Tehran, Iran; 4 Endocrine Research Center, Research Institute for Endocrine Sciences, Shahid Beheshti University of Medical Sciences, Tehran, Iran

**Keywords:** Cardiovascular Disease, Coronary Heart Disease, High-Density Lipoprotein, Subclass, HDL2-C, HDL3-C

## Abstract

**Background:**

The contribution of high-density lipoprotein cholesterol (HDL-C) subclasses to incident cardiovascular disease (CVD) and coronary heart disease (CHD) remains a subject of debate.

**Objectives:**

The objective of this study was to investigate these associations in a population with a high prevalence of dyslipidemia and CVD.

**Methods:**

In a nested case-control study, HDL-C and its subclasses (HDL2-C and HDL3-C) in 370 age and gender-matched case and control subjects were determined. This study employed multivariable-adjusted conditional logistic regression to calculate the odds ratios (ORs) for the associations between HDL-C, HDL2-C, HDL3-C, and HDL2-C/HDL3-C (both as continuous and categorical variables) with incident CVD and CHD. The present study models were adjusted for a comprehensive set of confounders, including body mass index, current smoking, hypertension, type 2 diabetes mellitus, use of lipid-lowering drugs, family history of premature CVD, non-HDL-C, and triglycerides.

**Results:**

In multivariate analysis, when considering lipoprotein parameters as continuous variables, a 1-unit increase in HDL-C and HDL3-C was associated with a reduced risk of incident CVD and CHD. For CVD, the ORs (95% confidence intervals [CI]) were 0.95 (0.92 - 0.98) and 0.95 (0.93 - 0.98) for HDL-C and HDL3-C, respectively. The corresponding values for CHD were 0.94 (0.91 - 0.97) and 0.94 (0.91 - 0.97). In the categorical approach to lipoprotein parameters, higher quartiles of HDL-C and HDL3-C, compared to the first quartile, were significantly associated with a lower risk of incident CVD and CHD. The ORs (95% CI) for the fourth quartiles were 0.43 (0.25 - 0.74, P for trend = 0.003) and 0.46 (0.27 - 0.78, P for trend = 0.005) for HDL-C and HDL3-C regarding CVD and 0.32 (0.17 - 0.59) and 0.32 (0.18 - 0.59) (all P for trend = 0.001) regarding CHD, respectively. Paradoxically, across quartiles of HDL2-C/HDL3-C, this lipid ratio was associated with a higher risk of CHD (92% higher risk in the fourth quartile).

**Conclusions:**

The results showed that HDL3-C, but not HDL2-C, was primarily responsible for the protective effect of HDL-C against CVD, particularly CHD, in Iranian adults.

## 1. Background

Cardiovascular disease (CVD) stands as one of the most significant public health concerns globally, accounting for approximately one-third of all worldwide mortality. The burden of CVD-related deaths has been on a steady rise, with numbers increasing from 12.1 million in 1999 to 18.6 million in 2019 (1, 2). Moreover, CVD constitutes the primary cause of both morbidity and mortality in the Eastern Mediterranean region (EMR) countries, including Iran (3). According to the Global Burden of Diseases (GBD) 2015 report, Iran has witnessed an incidence rate of more than 9 000 CVD cases per 100000 individuals, thereby ranking among the nations with the highest CVD rates globally (4).

Dyslipidemia is among the most prevalent risk factors for CVD in the EMR (3). Among the various forms of dyslipidemia, low high-density lipoprotein cholesterol (HDL-C) levels are notably common in the Iranian population, affecting over 73% of women and 64% of men (5, 6). Clinical and epidemiological studies have established a clear inverse relationship between HDL-C levels and the occurrence of CVD events, particularly coronary heart disease (CHD) (7, 8).

In comparison to other lipoproteins, HDL-C boasts a higher protein content primarily composed of apolipoprotein AI (Apo-A1), a component that plays a key role as an anti-inflammatory and anti-oxidative particle. Apolipoprotein AI also plays a pivotal role in reverse cholesterol transport (8, 9). High-density lipoprotein cholesterol, as a diverse lipoprotein family, comprises subclasses, with large HDL2-C and smaller, denser HDL3-C being key subclasses. These subclasses vary in size, density, shape, and lipid and apolipoprotein compositions, resulting in different structures, intravascular metabolism, and biological activities (8, 10, 11).

Despite the recognized athero- and cardioprotective roles of HDL-C, it was shown that the association between HDL-C levels and cardiovascular events is not consistent. Recent evidence from genetic and clinical research has recently challenged the longstanding belief that higher HDL-C levels consistently confer advantages whereas, low HDL-C levels always indicate increased risk (12). This issue underscores the complex association between HDL-C and CVD risk. Furthermore, in established cases of CHD, HDL-C levels have not consistently predicted the risk of major adverse cardiovascular events (9, 13). Studies on the association between HDL-C subclasses, particularly HDL2-C and HDL3-C, and incident CVD have yielded inconsistent results. Although some studies have reported significant relationships between both HDL2-C and HDL3-C levels and CVD (14-18), others have confined this association to HDL2-C (19-21) or HDL3-C (22-24).

To the best of our knowledge, no previous study has investigated the association between HDL-C subclasses and incident CVD in an Iranian population. The present study sought to explore the association between HDL2-C and HDL3-C and CVD within the framework of a nested case-control study conducted as part of the Tehran Lipid and Glucose Study (TLGS), the oldest cohort in the Middle East and North Africa (MENA) region.

## 2. Methods

### 2.1. Study Design

The TLGS is a community-based prospective cohort study conducted among the urban population of Tehran, Iran. The primary objectives of the study include determining the prevalence and incidence of non-communicable diseases (NCDs) and their associated risk factors among individuals aged ≥ 3 years. The study also aims to promote a healthy lifestyle to prevent NCDs. Participant enrollment for the TLGS occurred in two phases; the first phase took place between 1999 and 2001, involving 15 005 participants; nevertheless, the second phase spanned between 2001 and 2005 and included 3 550 individuals. The TLGS is planned to continue for at least 20 years, with tri-annual follow-up visits. Detailed information regarding the design and methodology of the TLGS has been previously published (25).

The current study employed a nested case-control design, comprising 740 individuals aged ≥ 30 years who participated in phase 3 of the TLGS and did not have CVD at the study’s commencement. The study population was divided into two groups: 370 patients who developed incident CVD during follow-up and 370 age and gender-matched control subjects randomly selected from the cohort.

The study protocol received approval from the Ethics Committee of Shahid Beheshti Medical University, Tehran, Iran, bearing approval number IR.SBMU.ENDOCRINE.REC.1399.104. Written informed consent was obtained from each participant.

### 2.2. Clinical and Laboratory Measurements

In the TLGS, qualified interviewers have been trained to collect participants’ information using a pretested questionnaire, including questions about demographics, history of CVD, medication, smoking habits, and family history of diabetes mellitus (FH-DM). The modifiable activity questionnaire (MAQ) was employed to assess physical activity levels, covering leisure time, job-related, and household activities over the past year (26). Anthropometric measurements were taken with participants wearing light clothing and without shoes. Details of anthropometric measurements have been previously reported (25). Body mass index (BMI) was calculated as weight (kg) divided by height (m²). Systolic and diastolic blood pressures (SBP and DBP) were measured on the right arm after a 15-minute resting period in a seated position, and the average of two measurements was recorded as the subject’s blood pressure.

All study participants fasted for 12 - 14 hours overnight, and blood samples were collected between 7:00 and 9:00 a.m. For the 2-hour post-challenge plasma glucose (2h-PCG) test, the participants orally received an 82.5 g glucose monohydrate solution (equivalent to 75 g anhydrous glucose), and blood samples were obtained 2 hours later.

Sample analyses were carried out using a Selectra 2 auto-analyzer (Vital Scientific, Spankeren, the Netherlands) and enzymatic colorimetric kits for total cholesterol (TC) and triglycerides (TG), employing cholesterol esterase and cholesterol oxidase for TC and glycerol phosphate oxidase for TG (Pars Azmon Inc., Tehran, Iran). High-density lipoprotein cholesterol was assessed using a direct enzymatic photometric method involving two steps; the first step was to eliminate non-HDL-C, followed by the measurement of HDL-C using cholesterol esterase and cholesterol oxidase (Delta Darman., Tehran, Iran).

Non-HDL cholesterol was calculated by subtracting HDL-C from TC. The intra- and inter-assay coefficients of variation (CVs) were 0.5% and 2.0% for TC, 0.6% and 1.6% for TG, and 0.7% and 1.8% for HDL-C, respectively. Glucose levels were assayed using an enzymatic colorimetric kit with glucose oxidase (Pars Azmon Inc., Tehran, Iran), with both intra- and inter-assay CVs of 2.2%.

### 2.3. Measuring HDL-C Subclasses Using A Single-Step Precipitation Method

The isolation of HDL-C subclasses was carried out through a single-step precipitation method according to the procedure described by Tsutomu Hirano (27). To precipitate HDL3-C, a single precipitant containing heparin, manganese chloride (MnCl2), and dextran sulfate (DS) reagent was employed, allowing for the simultaneous precipitation of both Apo-B containing lipoproteins and HDL2-C. The precipitation reagent was composed of 8.25 mg/mL heparin (Darou Pakhsh, Pharmaceutical Mfg. Co., Tehran, Iran; batch No. 339), 98.7 mg/mL MnCl2 (Merck KGaA, 64271 Darmstadt, Germany, Chemical Abstract Service 13446-34-9, catalog No. 105927), and 12 mg/mL DS (Sigma-Aldrich, St. Louis, MO; molecular weight 9000 - 20000, Chemical Abstract Service 9011-18-1; catalog No. D6924).

The precipitation process commenced by adding 0.04 mL of the reagent to 0.2 mL of serum. The final concentrations of the precipitant were 1.4 mg/mL heparin, 16.4 mg/mL MnCl2, and 2.0 mg/mL DS. After a 30-minute incubation at room temperature, the mixture underwent centrifugation at 10 000 rpm for 10 minutes at 4ºC, and the resulting supernatant was used for measuring HDL3-C. The measured value for total HDL3-C was multiplied by 1.2 (to account for dilution). High-density lipoprotein 2 cholesterol was calculated by subtracting HDL3-C from the directly determined total HDL-C. The intra- and inter-assay CVs were 1.0% and 2.9% for HDL3-C measurement.

### 2.4. Definitions

Prevalent CVD was defined as a self-reported history of specific types of CVD, including CHD or stroke. A positive family history of premature CVD was indicated by a prior diagnosis of CVD in first-degree male relatives aged < 55 and female relatives aged < 65 years.

Current smokers were individuals who smoked cigarettes or pipes daily or occasionally. Low physical activity was characterized by scores ≤ 600 metabolic equivalent tasks (MET)-minutes per week, based on the MAQ questionnaire (28). Hypertension was defined if SBP ≥ 140 mmHg or DBP ≥ 90 mmHg or if anti-hypertension medications were being used (29). Type 2 diabetes mellitus (T2DM) was identified by fasting plasma glucose (FPG) levels ≥ 126 mg/dL, 2-hour post-challenge plasma glucose (2h-PCG) levels ≥ 200 mg/dL, or the use of antidiabetic medications (30).

### 2.5. Outcomes

In the TLGS, a trained team annually followed up with the participants via phone calls to inquire about any medical conditions that had led to hospitalization in the previous year. Subsequently, a trained physician collected complementary data regarding that event during a home visit and by the acquisition of data from medical files. Finally, the outcome assessment committee of the TLGS, comprised of experts including an internist, endocrinologist, cardiologist, pathologist, epidemiologist, and others, assessed the gathered data to assign specific outcomes to each event. In this study, CHD events encompassed a composite measure of definite and probable myocardial infarction (MI), unstable angina, angiographically proven CHD and CHD-related deaths. Cardiovascular disease was defined as a composite of CHD, possible stroke, transient ischemic attack, or cerebrovascular-related deaths.

### 2.6. Statistical Analyses

Baseline characteristics were presented as mean (standard deviation [SD]) for continuous variables with normal distribution, median (interquartile range [IQR]) for skewed continuous variables, and frequencies (%) for categorical variables. To compare baseline characteristics between participants with incident CVD and those without the event, the student’s *t*-test and the Mann-Whitney test were employed for continuous variables; however, the chi-square test was used for categorical variables. Normality was assessed using the Kolmogorov-Smirnov (K-S) test, and histograms with fitted normal curves were also examined to verify data normality.

Multivariable conditional logistic regression analysis was conducted to assess the association between HDL-C, HDL2-C, HDL3-C, and HDL2-C/HDL3-C as continuous (per 1 unit increase) and as quartile measures (the first quartile as reference). Odds ratios (ORs) with 95% confidence intervals (CIs) were reported in three following models:

- Model 1: Adjusted for BMI, current smoking, hypertension, T2DM, use of lipid-lowering drugs, family history of premature CVD, and low physical activity

- Model 2: Further adjusted for non-HDL-C

- Model 3: Further adjusted for TG

The analyses were carried out using SPSS for Windows version 20, STATA version 14 SE (Stata Corp LP, TX, USA), and R software, with a significance level set at P < 0.05.

## 3. Results

The study population included 370 cases and 370 age- and gender-matched control subjects, with a mean age (SD) of 57.1 (11.3) years and a median (IQR) follow-up duration of 11.6 (10.7 - 12.2) years. Table 1 shows the baseline characteristics and a comparison between cases and controls.

**Table 1.** Baseline Characteristics of the Study Population: Tehran Lipid and Glucose Study

Variables	Without CVD (Controls) n = 370	With CVD (Cases) n = 370	P-Value
**Continuous Variables; mean (SD)**			
**Age (y)**	57.1 (11.3)	57.1 (11.3)	0.997
**SBP (mmHg)**	122.6 (17.9)	129.0 (20.5)	< 0.001
**DBP (mmHg)**	76.3 (10.5)	79.1 (11.0)	< 0.001
**FPG (mmol/L)**	5.76 (1.96)	6.26 (2.56)	0.003
**2h-PCG (mmol/L)**	6.94 (3.39)	6.94 (3.14)	0.974
**BMI (kg/mm** ^**2**^ **)**	28.6 (4.6)	28.7 (4.6)	0.651
**TC (mmol/L)**	5.22 (1.01)	5.39 (1.07)	0.025
**TG (mmol/L)**	1.97 (1.27)	2.26 (1.60)	0.007
**Non-HDL-C (mmol/L)**	4.11 (0.93)	4.31 (0.99)	0.004
**HDL-C (mmol/L)**	1.11 (0.18)	1.08 (0.16)	0.027
**HDL2-C (mmol/L) **	0.34 (0.10)	0.35 (0.10)	0.059
**HDL3-C (mmol/L)**	0.77 (0.18)	0.73 (0.16)	0.001
**HDL2-C/HDL3-C ratio**	0.47 (0.20)	0.51 (0.21)	0.006
**Categorical Variables; No. (%)**			
**Gender**			1.000
Male	202 (54.6)	202 (54.6)	
Female	168 (45.4)	168 (45.4)	
**Family history of premature CVD, yes**	89 (24.1)	114 (30.8)	0.039
**Smoking**			0.133
Never	294 (79.5)	271 (73.2)	
Past	33 (8.9)	45 (12.2)	
Current	43 (11.6)	54 (14.6)	
**Hypertension, yes**	100 (27.0)	140 (37.8)	0.002
**T2DM, yes**	51 (13.8)	84 (22.7)	0.002
**Low physical activity**	226 (61.1)	233 (63.0)	0.596
**Lipid-lowering drugs, yes**	29 (7.8)	31 (8.4)	0.788
**Anti-diabetic medication**	31 (8.4)	54 (14.6)	0.008
**Anti-hypertension drugs**	30 (8.1)	44 (11.9)	0.086

Abbreviations: SBP, systolic blood pressure; DBP, diastolic blood pressure; FPG, fasting plasma glucose; 2h-PCG, 2-hour post-challenge plasma glucose; BMI, body mass index; TC, total cholesterol; TG, triglycerides; HDL-C, high density lipoprotein cholesterol; CVD, cardiovascular disease; T2DM, type 2 diabetes mellitus; SD, standard deviation.

**Figure 1.**
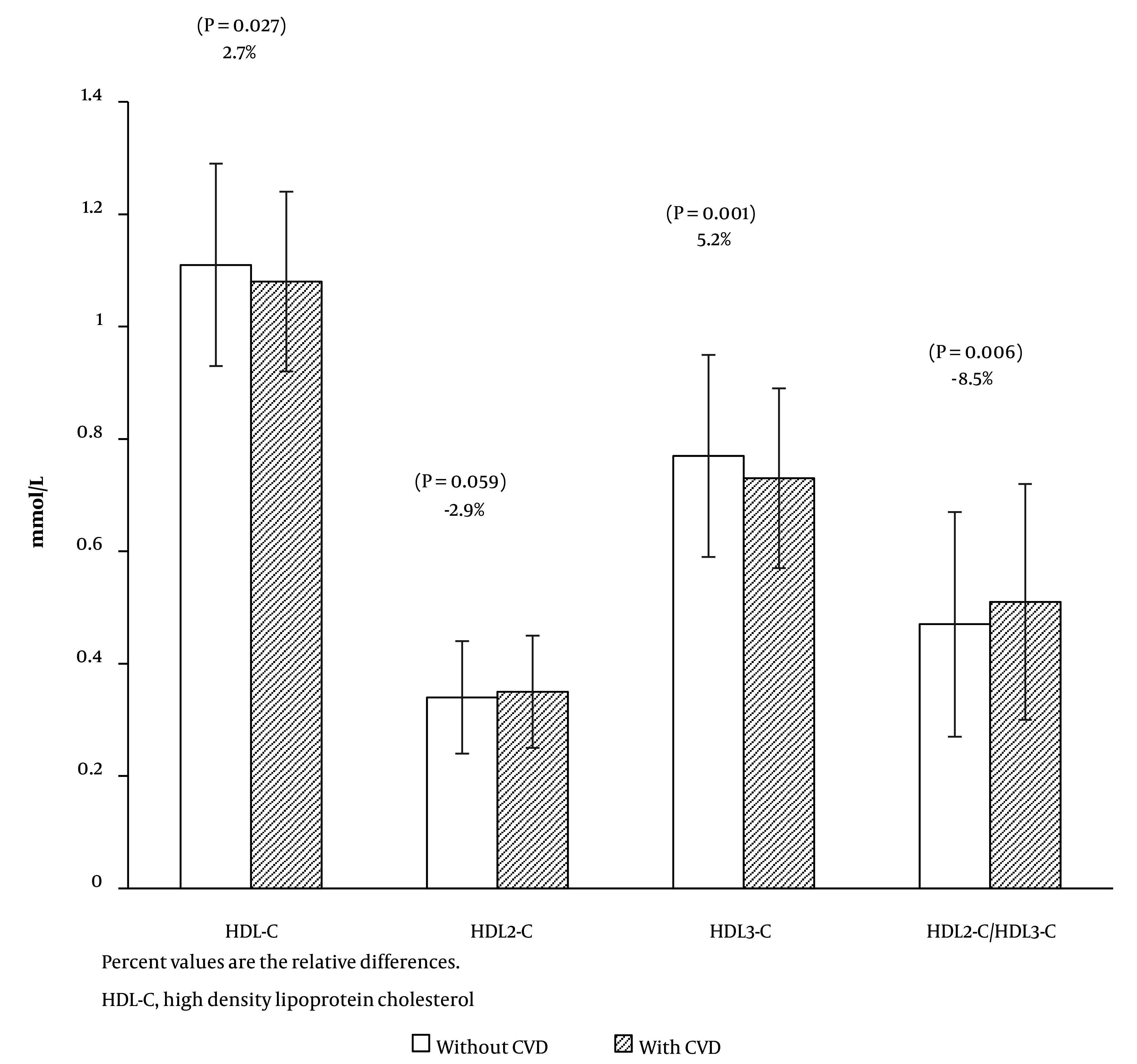
Comparison of the mean (standard deviation) of HDL-C, HDL2-C, HDL3-C, and HDL2-C/HDL3-C at baseline between cardiovascular disease (−) and (+) subjects.

Participants with incident CVD exhibited higher levels of SBP, DBP, and FPG, along with a higher frequency of family history of premature CVD. Additionally, individuals in the case group were more likely to have hypertension and T2DM, and use anti-diabetic medication. Regarding lipid measures, when compared to participants without incident CVD, the case group had significantly higher levels of TC, TG, non-HDL-C, and HDL2-C/HDL3-C; nevertheless, their levels of HDL-C and HDL3-C were lower. High-density lipoprotein 3 cholesterol constituted 69.4% and 67.6% of HDL-C in the control and case groups, respectively. Figure 1 illustrates the comparison of baseline HDL-C, HDL2-C, HDL3-C, and HDL2-C/HDL3-C, along with their relative differences between cases and controls.

Table 2 shows the results of the multivariable conditional logistic regression analysis, which examined the ORs with 95% CIs for HDL-C and its subclasses as continuous variables in relation to incident CVD and CHD. For CVD, in crude and all three adjusted models, HDL-C and HDL3-C were associated with lower risk; the ORs (95% CIs) for model 3 were [0.95 (0.92 - 0.98)] and [0.95 (0.93 - 0.98)], respectively. Although the HDL2-C/HDL3-C was significantly associated with incident CVD in the crude and model 1, further adjustment for non-HDL-C significantly attenuated this association. Considering CHD outcome, similar to CVD, HDL-C and HDL3-C were associated with incident CHD in all models; the ORs (95% CIs) for model 3 were [0.94 (0.91 - 0.97)] and [0.94 (0.91 - 0.97)], respectively. On the other hand, although HDL2-C and HDL2-C/HDL3-C showed associations with incident CHD in the crude and models 1 and 2, these associations disappeared in model 3.

**Table 2.** Odds Ratios (95% Confidence Intervals) Per 1 Unit Increase from Multivariable Analysis of Cardiovascular Disease and Coronary Heart Disease Across High-Density Lipoprotein Cholesterol Subclasses

Variables	Crude Model		Model 1 ^a^		Model 2 ^b^		Model 3 ^c^	
	OR (95% CI)	P-Value	OR (95% CI)	P-Value	OR (95% CI)	P-Value	OR (95% CI)	P-Value
**CVD**								
**HDL-C**	0.97 (0.94 - 0.99)	0.016	0.97 (0.95 - 1.00)	0.023	0.95 (0.92 - 0.98)	< 0.001	0.95 (0.92 - 0.98)	< 0.001
**HDL2-C**	1.03 (0.99 - 1.07)	0.066	1.03 (0.99 - 1.07)	0.127	0.99 (0.94 - 1.04)	0.718	0.98 (0.94 - 1.04)	0.555
**HDL3-C**	0.95 (0.93 - 0.97)	< 0.001	0.96 (0.93 - 0.98)	0.001	0.95 (0.93 - 0.98)	0.001	0.95 (0.93 - 0.98)	0.001
**HDL2-C/HDL3-C **	3.14 (1.44 - 6.85)	0.004	2.70 (1.21 - 6.05)	0.015	1.79 (0.76 - 4.19)	0.182	1.67 (0.68 - 4.10)	0.266
**CHD**								
**HDL-C**	0.96 (0.93 - 0.99)	0.010	0.96 (0.94 - 0.99)	0.011	0.94 (0.91 - 0.97)	< 0.001	0.94 (0.91 - 0.97)	< 0.001
**HDL2-C**	1.04 (1.00 - 1.09)	0.045	1.05 (1.00 - 1.10)	0.062	1.00 (0.94 - 1.06)	0.980	0.99 (0.93 - 1.05)	0.780
**HDL3-C**	0.94 (0.91 - 0.97)	< 0.001	0.94 (0.91 - 0.97)	< 0.001	0.94 (0.91 - 0.97)	< 0.001	0.94 (0.91 - 0.97)	< 0.001
**HDL2-C/HDL3-C**	4.27 (1.74 - 10.47)	0.002	4.09 (1.61 - 10.37)	0.003	2.62 (0.97 - 7.09)	0.058	2.44 (0.86 - 6.94)	0.095

Abbreviations: OR, odds ratio; CI, confidence interval; CVD, cardiovascular disease; CHD, coronary heart disease; HDL-C, high-density lipoprotein cholesterol.

^a^ Model 1: Adjusted for body mass index, current smoking, hypertension, type 2 diabetes mellitus, lipid-lowering drugs, family history of cardiovascular and low physical activity.

^b^ Model 2: Model 1 + non-HDL-C.

^c^ Model 3: Model 2 + triglycerides.

Conditional logistic regression analyses were also conducted to further assess the associations between quartiles of HDL-C its subclasses and incident CVD and CHD. As detailed in Table 3, HDL-C exhibited an association with incident CVD in all models, with statistical significance in both models 2 (P for trend = 0.002) and model 3 (P for trend = 0.003) across all quartiles. Notably, individuals in the highest quartile of HDL3-C (≥ 0.85 mmol/L) experienced a 54% reduced risk of incident CVD. However, it is worth mentioning that no significant relationship was observed between increasing levels of HDL2-C and incident CVD, even in the crude model. Regarding HDL2-C/HDL3-C, the association with increasing levels of this ratio was only evident in the crude model and model 1.

**Table 3.** Association of High-Density Lipoprotein Cholesterol Subclasses with the Incidence of Cardiovascular Disease: Tehran Lipid and Glucose Study ^a, b^

Variables	Quartile 2		Quartile 3		Quartile 4		
	OR (95% CI)	P-Value	OR (95% CI)	P-Value	OR (95% CI)	P-Value	P for Trend
HDL-C (mmol/L)	0.98 ≤ HDL-C < 1.08		1.08 ≤ HDL-C < 1.20		1.20 ≤ HDL-C		
**Crude**	0.74 (0.48 - 1.13)	0.173	0.71 (0.46 - 1.09)	0.125	0.63 (0.40 - 1.00)	0.052	0.061
**Model 1**	0.72 (0.46 - 1.13)	0.158	0.68 (0.43 - 1.07)	0.095	0.65 (0.40 - 1.04)	0.075	0.079
**Model 2**	0.58 (0.36 - 0.93)	0.023	0.52 (0.32 - 0.84)	0.008	0.43 (0.25 - 0.72)	0.002	0.002
**Model 3**	0.58 (0.36 - 0.94)	0.027	0.52 (0.32 - 0.86)	0.010	0.43 (0.25 - 0.74)	0.002	0.003
**HDL2-C (mmol/L)**	**0.28 ≤ HDL2-C < 0.34**		**0.34 ≤ HDL2-C < 0.40**		**0.40 ≤ HDL2-C**		
**Crude**	0.96 (0.63 - 1.48)	0.885	1.12 (0.72 - 1.72)	0.599	1.33 (0.88 - 2.00)	0.169	0.123
**Model 1**	0.95 (0.61 - 1.47)	0.817	1.03 (0.66 - 1.62)	0.888	1.24 (0.81 - 1.90)	0.322	0.279
**Model 2**	0.80 (0.50 - 1.26)	0.332	0.77 (0.47 - 1.25)	0.292	0.78 (0.46 - 1.30)	0.335	0.360
**Model 3**	0.79 (0.50 - 1.26)	0.323	0.76 (0.47 - 1.24)	0.276	0.72 (0.42 - 1.23)	0.228	0.240
**HDL3-C (mmol/L)**	**0.62 ≤ HDL3-C < 0.73**		**0.73 ≤ HDL3-C < 0.85**		**0.85 ≤ HDL3-C**		
**Crude**	0.80 (0.52 - 1.21)	0.300	0.81 (0.52 - 1.24)	0.343	0.47 (0.29 - 0.75)	0.002	0.003
**Model 1**	0.88 (0.57 - 1.36)	0.576	0.81 (0.52 - 1.27)	0.358	0.50 (0.31 - 0.81)	0.005	0.006
**Model 2**	0.79 (0.51 - 1.24)	0.304	0.72 (0.45 - 1.15)	0.170	0.47 (0.29 - 0.77)	0.003	0.001
**Model 3**	0.79 (0.50 - 1.24)	0.302	0.72 (0.44 - 1.15)	0.171	0.46 (0.27 - 0.78)	0.004	0.005
**HDL2-C/HDL3-C **	**0.34 ≤ HDL2-C/HDL3-C < 0.46**		**0.46 ≤ HDL2-C/HDL3-C < 0.60**		**0.60 ≤ HDL2-C/HDL3-C**		
**Crude**	1.35 (0.74 - 2.49)	0.320	1.65 (0.88 - 3.09)	0.111	2.17 (1.17 - 4.01)	0.013	0.003
**Model 1**	1.20 (0.77 - 1.86)	0.420	1.33 (0.85 - 2.09)	0.209	1.74 (1.14 - 2.65)	0.010	0.009
**Model 2**	1.12 (0.72 - 1.75)	0.612	1.15 (0.72 - 1.83)	0.571	1.40 (0.89 - 2.21)	0.142	0.149
**Model 3**	1.12 (0.72 - 1.75)	0.625	1.13 (0.70 - 1.81)	0.610	1.36 (0.84 - 2.20)	0.207	0.225

Abbreviations: HDL-C, high-density lipoprotein cholesterol; CVD, cardiovascular disease; OR, odds ratio; CI, confidence interval.

^a^ The first quartile of each subclass was considered the reference group (HDL-C < 0.98 mmol/L, HDL2-C < 0.28 mmol/L, HDL3-C < 0.62 mmol/L, and HDL2-C/HDL3-C < 0.34).

^b^ Model 1: Adjusted for body mass index, current smoking, hypertension, type 2 diabetes mellitus, lipid-lowering drugs, family history of cardiovascular and low physical activity; Model 2: Model 1 + non-HDL-C; Model 3: Model 2 + triglycerides.

Table 4 shows the ORs with their corresponding 95% CIs for quartiles of HDL-C and its subclasses and incident CHD. Individuals in the highest quartile of HDL-C exhibited a 68% lower risk of CHD (P for trend = 0.001). In terms of the association between HDL-C subclasses and incident CHD, it was observed that even after adjusting for all confounding factors, higher quartiles of HDL-C and HDL3-C were significantly linked to a reduced risk (P for trends = 0.001). Paradoxically, when examining quartiles of HDL2-C/HDL3-C, this lipid ratio was associated with an elevated risk of CHD, although this trend tended to be significant (P = 0.072). Notably, the fourth quartile of HDL2-C/HDL3-C was associated with a 92% higher risk of CHD.

**Table 4.** Association of High-Density Lipoprotein Cholesterol Subclasses with the Incidence of Coronary Heart Disease: Tehran Lipid and Glucose Study ^a, b^

Variables	Quartile 2		Quartile 3		Quartile 4		
	OR (95% CI)	P-Value	OR (95% CI)	P-Value	OR (95% CI)	P-Value	P for Trend
HDL-C (mmol/L)	0.98 ≤ HDL-C < 1.08		1.08 ≤ HDL-C < 1.19		1.19 ≤ HDL-C		
**Crude**	0.53 (0.32 - 0.87)	0.12	0.56 (0.34 - 0.92)	0.023	0.52 (0.31 - 0.87)	0.014	0.025
**Model 1**	0.51 (0.30 - 0.85)	0.011	0.51 (0.30 - 0.86)	0.011	0.52 (0.30 - 0.89)	0.016	0.027
**Model 2**	0.37 (0.21 - 0.65)	0.001	0.36 (0.20 - 0.64)	< 0.001	0.32 (0.17 - 0.58)	< 0.001	0.001
**Model 3**	0.37 (0.21 - 0.66)	0.001	0.36 (0.20 - 0.65)	0.001	0.32 (0.17 - 0.59)	< 0.001	0.001
**HDL2-C (mmol/L)**	**0.28 ≤ HDL2-C < 0.33**		**0.33 ≤ HDL2-C < 0.40**		**0.40 ≤ HDL2-C**		
**Crude**	0.86 (0.54 - 1.38)	0.554	0.96 (0.59 - 1.56)	0.890	1.35 (0.85 - 2.14)	0.201	0.158
**Model 1**	0.86 (0.53 - 1.40)	0.544	0.90 (0.54 - 1.49)	0.676	1.30 (0.80 - 2.10)	0.287	0.252
**Model 2**	0.70 (0.42 - 1.17)	0.175	0.65 (0.37 - 1.12)	0.122	0.76 (0.42 - 1.38)	0.368	0.394
**Model 3**	0.69 (0.42 - 1.16)	0.163	0.64 (0.37 - 1.12)	0.118	0.70 (0.38 - 1.29)	0.256	0.258
**HDL3-C (mmol/L)**	**0.61 ≤ HDL3-C < 0.73**		**0.73 ≤ HDL3-C < 0.84**		**0.84 ≤ HDL3-C**		
**Crude**	0.65 (0.40 - 1.04)	0.077	0.60 (0.36 - 0.99)	0.047	0.38 (0.22 - 0.65)	< 0.001	0.001
**Model 1**	0.65 (0.40 - 1.65)	0.081	0.58 (0.35 - 0.98)	0.041	0.36 (0.20 - 0.63)	< 0.001	< 0.001
**Model 2**	0.57 (0.34 - 0.94)	0.027	0.53 (0.31 - 0.90)	0.018	0.34 (0.19 - 0.60)	< 0.001	< 0.001
**Model 3**	0.55 (0.33 - 0.93)	0.026	0.51 (0.30 - 0.89)	0.017	0.32 (0.18 - 0.59)	< 0.001	0.001
**HDL2-C/HDL3-C **	**0.35 ≤ HDL2-C/HDL3-C < 0.46**		**0.46 ≤ HDL2-C/HDL3-C < 0.60**		**0.60 ≤ HDL2-C/HDL3-C**		
**Crude**	1.31 (0.65 - 2.66)	0.438	1.74 (0.84 - 3.58)	0.129	2.22 (1.09 - 4.52)	0.026	0.001
**Model 1**	1.23 (0.76 - 1.99)	0.400	1.45 (0.93 - 2.29)	0.104	2.52 (1.44 - 4.39)	0.001	0.002
**Model 2**	1.16 (0.71 - 1.90)	0.548	1.25 (0.78 - 2.02)	0.354	1.98 (1.09 - 3.60)	0.026	0.042
**Model 3**	1.16 (0.71 - 1.89)	0.552	1.24 (0.76 - 2.01)	0.392	1.92 (1.02 - 3.61)	0.043	0.072

Abbreviations: HDL-C, high-density lipoprotein cholesterol; CHD, coronary heart disease; OR, odds ratio; CI, confidence interval.

^a^ The first quartile of each subclass was considered the reference group (HDL-C < 0.98 mmol/L, HDL2-C < 0.28 mmol/L, HDL3-C < 0.61 mmol/L, and HDL2-C/HDL3-C < 0.35).

^b^ Model 1: Adjusted for body mass index, current smoking, hypertension, type 2 diabetes mellitus, lipid-lowering drugs, family history of cardiovascular and low physical activity; Model 2: Model 1 + non-HDL-C; Model 3: Model 2 + triglycerides.

## 4. Discussion

The current study explored potential associations between HDL-C and its subclasses with incident CVD and CHD within the Iranian population. The obtained findings revealed significant inverse relationships between HDL-C and HDL3-C levels and incident CVD and CHD, even after adjusting for a comprehensive set of confounders, including non-HDL-C and TG. Notably, these associations were more pronounced for CHD. Moreover, we found a signal that the increasing value of HDL2-C/HDL3-C was accompanied by a higher risk of CHD, as the 4th quartile (ratio ≥ 0.60) had more than 90% higher risk for the event. However, no significant association was observed between HDL2-C and our outcomes.

Historically, the use of different techniques and procedures, in addition to the consideration of HDL’s physicochemical and functional properties, has led to varying terms for defining HDL species. The two main subclasses, HDL2 and HDL3, are categorized based on their lipid-to-protein ratio and are to large and small HDL particles (31). Several studies have assessed the associations between cholesterol levels in these lipoprotein subclasses and the risk of CVD, yielding inconsistent results (15, 21, 32). These conflicting findings might be attributed to ethnic diversity or differing study designs, including variations in inclusion and exclusion criteria, outcome definitions, levels of adjustment for confounders, and methods employed for determining HDL-C subclasses.

In the current investigation, it was determined that the protective effect of HDL-C could be primarily attributed to HDL3-C. Yu et al., in a prospective cohort study, evaluated the relationship between HDL-C and its subclasses and CHD risk in British men. Yu et al. identified significant inverse associations between HDL-C and HDL3-C, but not HDL2-C, with incident CHD. However, it is important to note that the researchers adjusted but did not exclude pre-existing CHD in their data analysis (22). In a meta-analysis by Joshi et al., which included data from two American cohorts, it was reported that the denser HDL3-C subclass is the major determinant of the inverse association between HDL-C and incident CHD, with a multivariate hazard ratio (HR) of 0.75 (95% CI: 0.92 - 0.94); nevertheless, HDL2-C showed no significant association with CHD risk (32). Similarly, in the present data analysis, a 1-unit increase in HDL3-C was associated with a 6% lower risk of CHD. Furthermore, findings from the Ludwigshafen Risk and Cardiovascular Health (LURIC) study suggested that high concentrations of total HDL particles were inversely related to cardiovascular mortality, and this relationship was mediated by small HDL particles, with multivariate HRs of 0.55 (0.42 - 0.72) for total HDL and 0.60 (0.46 - 0.77) for small HDL particles (33). In the Hong Kong Diabetes Biobank (HKDB) prospective cohort study, Jin et al. examined the relationship between HDL subclasses and cardiovascular outcomes in patients with T2DM and observed that small HDL particles had an inverse correlation with the development of CVD and all-cause mortality (34).

The association of HDL-C subclasses with CVD/CHD has also been assessed as a predictive tool in secondary prevention. Albers et al. reported that higher baseline levels of HDL3-C, but not HDL2-C, were associated with fewer adverse events in patients with established CVD who received intensive low-density lipoprotein cholesterol (LDL-C) lowering therapy with statins (24). Martin et al. collaboratively analyzed the data from two prospective cohort studies to assess the association of HDL2-C and HDL3-C with clinical outcomes in patients with established CHD. Their results demonstrated that lower levels of HDL3-C, but not HDL2-C, were strongly associated with higher adverse outcomes (23).

In contrast to the findings of the present study, the protective role of HDL2-C regarding CVD events has been addressed in other studies (20, 21, 35). In the Kuopio study, researchers showed that HDL2-C, but not HDL3-C, which constituted about 65% of total HDL-C, was associated with a lower risk of MI among Finnish men (19). Another cohort study conducted on French-Canadian men reported that although the fourth quartiles of HDL-C and its subclasses were associated with a reduction in the risk of incident ischemic heart disease, the magnitude of this risk reduction was higher for HDL2-C. Specifically, men in the highest quartile of HDL2-C had a 4.8-fold decreased risk compared to those in the first quartile (21).

Li et al., in a prospective observational cohort study of Chinese subjects with stable coronary artery disease (CAD), showed that high levels of large HDL-C particles were inversely associated with several traditional risk factors, in addition to the severity of CAD at baseline and the incidence of major adverse cardiovascular events (36). In a study conducted on participants in the Prospective Multicenter Imaging Study for Evaluation of Chest Pain (PROMISE) trial, it was shown that there is an inverse association between large and medium HDL subclasses and high-risk coronary atherosclerotic plaque features. Additionally, the study showed that medium HDL subclasses had an inverse association with major adverse cardiovascular events (37).

In the current study, it was observed that an increasing value of HDL2-C/HDL3-C was associated with CHD events, and this trend tended to be significant. Few studies have examined the association of HDL-C subclass ratios with CVD. A study in a Japanese population assessed the relationship between HDL2-C/HDL3-C and CVD risk factors, showing that a higher ratio was inversely associated with changes in waist circumference, insulin resistance, and LDL-C, and favorably associated with changes in healthy lifestyle habits, including smoking status, exercise, physical activity, and alcohol consumption (38).

Several potential mechanisms might play roles in the association between HDL-C subclasses and incident CVD. The current study findings can be explained through the distinct biological functions of HDL subclasses. High-density lipoprotein 3, which includes smaller, denser particles enriched in bioactive lipids and proteins, has a more potent cholesterol efflux capacity compared to other HDL subclasses (39, 40), with an 8.02% efflux capacity for HDL3c compared to 4.48% for HDL2b (39). Joshi et al. previously suggested that the higher cholesterol content within denser HDL3 particles might represent a more competent reverse cholesterol transport system (32).

Furthermore, considering their anti-oxidative, anti-inflammatory, anti-thrombotic, and anti-apoptotic properties, small, dense HDL particles are more potent than HDL2-C. In addition, small, dense HDL3 contains not only a higher quantity of proteins but also a greater variety of distinct, functional proteins than large, light HDL2 (39). In addition, considering bioactive lipids, the content of sphingosine-1-phosphate (S1P), a minor bioactive lysosphingolipid, is elevated in small, dense HDL, whereas that of sphingomyelin (SM) is reduced, leading to increased S1P/SM molar ratio in HDL3. The S1P/SM molar ratio has been reported to strongly and positively correlate with the anti-apoptotic and anti-oxidative activities of HDL subclasses. Sphingosine-1-phosphate contributes to HDL-mediated protection from apoptosis and can explain the cytoprotective role of HDL3. Furthermore, small HDL3 effectively attenuated endothelial cell apoptosis and delayed LDL oxidation (39, 41).

As a strength, the current study was conducted as a nested case-control study within the context of a prospective cohort in a region with a high burden of CVD. Other strengths included the high-quality protocol for the assessment of clinical and outcome data and the performance of all laboratory parameters, including HDL-C subclasses, in a single laboratory by expert staff. Furthermore, to the best of our knowledge, this study was the first population-based study to examine the association of HDL-C subclasses with incident CVD in the Iranian population. However, this study has some limitations. Firstly, HDL-C subclasses were measured using a non-reference method; however, this is the case in some similar studies (16, 17, 21, 22), and it has been reported that the single-step precipitation method correlates excellently with the reference method (ultracentrifugation) (27). Secondly, using a single baseline measurement of HDL-C and its subclasses precluded the consideration of the effects of changes in these lipid parameters during the follow-up period. Finally, as the current study was conducted in the metropolitan city of Tehran, its results might not be extrapolatable to rural regions.

### 4.1. Conclusions

This study demonstrated that HDL3-C, but not HDL2-C, was mainly responsible for the protective impact of HDL-C against CVD, especially CHD, among Iranian adults. Moreover, it was demonstrated that increasing the value of HDL2-C/HDL3-C was associated with a higher risk of CHD.

## Data Availability

The dataset presented in the study is available on request from the corresponding author during submission or after publication.
